# Effect of Drying Technique on the Volatile Content of Ecuadorian Bulk and Fine-Flavor Cocoa

**DOI:** 10.3390/foods12051065

**Published:** 2023-03-02

**Authors:** Cyntia Yadira Erazo Solorzano, Vincenzo Disca, José Manuel Muñoz-Redondo, Diego Armando Tuárez García, Mónica Sánchez-Parra, Manuel Danilo Carrilo Zenteno, José Manuel Moreno-Rojas, Raquel Rodríguez-Solana

**Affiliations:** 1 Faculty of Industry and Production Sciences, State Technical University of Quevedo, Av. Walter Andrade, km 1.5 Via Santo Domingo, Quevedo 120301, Ecuador; 2 Dipartimento di Scienze del Farmaco, Università degli Studi del Piemonte Orientale “A. Avogadro”, 28100 Novara, Italy; 3 Department of Agrifood Industry and Food Quality, Andalusian Institute of Agricultural and Fisheries Research and Training (IFAPA), Alameda del Obispo, Avda Menéndez Pidal s/n, 14004 Córdoba, Spain; 4 Instituto Nacional de Investigaciones Agropecuarias (INIAP), Estación Experimental Tropical Pichilingue (EETP), Departamento de Manejo de Suelos y Aguas (DMSA), km 5 Via Quevedo–El Empalme, Cantón Mocache, Quevedo 120313, Ecuador; 5 Foods for Health Group, Instituto Maimónides de Investigación Biomédica de Córdoba (IMIBIC), 14004 Córdoba, Spain; 6 MED—Mediterranean Institute for Agriculture, Environment and Development & CHANGE—Global Change and Sustainability Institute, Faculdade de Ciências e Tecnologia, Universidade do Algarve, Campus de Gambelas, 8005-139 Faro, Portugal

**Keywords:** *Theobroma cacao* L., drying techniques, HS-SPME-GC-MS, volatiles, multivariate analysis

## Abstract

Cocoa (*Theobroma cacao* L.) is one of the most widely consumed edible seeds in the world affected by on-farm processing. This study investigated the effect of different drying techniques, namely oven drying (OD), sun drying (SD), and a modification of sun drying using black plastic sheeting (SBPD), on the volatile profile of fine-flavor and bulk cocoa varieties analyzed by HS-SPME-GC-MS. A total of sixty-four volatile compounds were identified in fresh and dried cocoa. As expected, the volatile profile was clearly modified after the drying step, showing strong differences among cocoa varieties, this factor and its interaction with the drying technique having greater influence according to the ANOVA simultaneous component analysis. A principal component analysis revealed a close relationship between the volatile content of bulk cocoa samples dried by the OD and SD techniques, whereas slight volatile modifications were perceived among fine-flavor samples dried using the three different techniques under study. Overall, the results provide the basis for the potential application of the simplest inexpensive SBPD technique in order to accelerate the drying process of sun drying and produce cocoa with similar (fine-flavor cocoa) or improved (bulk cocoa) aromatic quality to that formed using the traditional SD or the small-scale OD.

## 1. Introduction

Cocoa (*Theobroma cacao* L.) beans are an important global raw material for a multitude of industries including foods and beverages, cosmetics, and pharmaceuticals [1]. According to the Observatory of Economic Complexity (OEC), cocoa beans represent 0.051% of the total world trade, with a total trade of 8.54 billion U.S. dollars [2].

The planting and harvesting of cocoa beans, as well as the on-farm (primary) post-harvest treatments, namely fermentation and drying, are carried out in the countries of origin. Both primary processes have a complementary and important influence on bean quality, since some chemical changes (development of the right color, flavor, and aroma) produced during fermentation continue along the drying process [3]. This process is one of the oldest and most-efficient food processes used to preserve samples. In the case of cocoa drying, the bean moisture content is reduced from ca. 60% to 8–5%, a moisture level that can be considered safe for prolonged storage and suitable for marketing before distribution to the manufacturing areas of cocoa products. Therefore, the good proceeding of both primary processes produces samples with optimal properties to be transformed in the secondary or industrial processing countries [4].

Thus, the choice of the drying technique is an important issue strongly dependent on different factors such as the regional weather conditions, the cocoa genotype, and the economic conditions of each farmer [1]. It is also important to consider that each type of technique has associated characteristics that can affect the final cocoa quality.

In this sense, in terms of suitability and possible usage for large-scale drying by local farmers (smallholders), the natural or open sun drying technique (conventional) is the oldest passive mechanism. This technique is the most popular in the tropical and subtropical areas where the solar radiation is abundant during the harvest season [5]. It is a non-polluting (environmentally friendly), freely available technique and the most preferred due to its simplicity (using rudimentary procedures) and low construction and running costs. Furthermore, this type of intermittent drying technique allows the slow migration of moisture throughout the beans, which transports the flavor precursors formed during fermentation. However, some of the drawbacks found are based on it being a labor-intensive technique and weather-dependent. Thus, the exposure of the beans to long drying periods (7–22 days) can result in mold growth and the appearance of different environmental contaminants [6]. This increases the risk of resulting in poor-quality beans with a lower price on the market compared to other drying techniques with a more controlled process.

The artificial drying techniques are necessary in several countries such as Cameroon, Costa Rica, Malaysia, Fernando Pó, Panama, and Brazil, because the harvesting period coincides with frequent rainfall weather periods [1]. Examples of artificial drying techniques are electric (oven and dehydrators), diesel- and wood-fired dryers, and microwave and freeze dryers. Among the artificial techniques, firewood oven dryers are commonly used because of the easy usage and relatively low cost. However, their main disadvantage lies in the difficulty to control the smoke, which transfers undesirable aromas and flavors to the dried cocoa, and to maintain a constant temperature (35–40 °C) and distribution of the heat [1,7]. Other artificial dryers less commonly used are electric dehydrators, oven drying (laboratory setting type of low-temperature convection or forced air oven, where the moisture is removed evenly and slowly), microwave drying (produces a rapid energy coupling into the moisture and leads to fast heating and drying), and freeze drying (frozen water in the food turns to vapor by sublimation and is eliminated by a strong vacuum). Despite their great economy of time and general effectiveness in eliminating moisture and producing a homogeneous and controlled drying process, most of these dryers produce quick-dried or even over-dried cocoa [8]. This leads to the hardening of the shell, causing the entrapment or locking of the volatile acids inside the bean, obtaining a high-acid and low-chocolate-flavored cocoa character. Therefore, the knowledge of the drying rate (temperature and air velocity) is very important in predicting the drying time of biomaterials, where lower air temperatures or an overnight rest period can equilibrate the moisture in the beans, preventing the abovementioned consequences [9]. Moreover, small wet bean loads (<50 kg/m^2^) are recommended to avoid mold contamination. Furthermore, these techniques present reduced spaces for the large-scale drying and high-energy consumption (high electrical and technical inputs) inherent to the equipment used.

In recent times, the search for cleaner, sustainable, environmentally friendly, less expensive, and more efficient technologies is a great challenge for farmers, in order to counter the less efficient characteristics of conventional techniques. The search for sustainable slight variations in conventional techniques could be a good option. To date, there are an extensive number of studies related to the influence of the drying techniques and conditions on the chemical composition of the cocoa quality [1,3,4,6,9]. However, the current study proposes, for the first time, the study of the influence and comparison of two large-scale techniques, the traditional sun drying (SD) technique and a modification of this technique using black plastic over a concrete floor (SBPD) as a sustainably affordable alternative technology to the traditional and more accessible mentioned technique, as well as artificial oven drying (OD), to evaluate their effect on the volatile composition of the preconditioned (pre-dried) cocoa from two types of cocoa varieties: Ecuadorian bulk (Forastero and CCN-51) and high-grade fine-flavor (ETT-103 and LR-14) cocoa. To address this, the chromatographic data were evaluated by means of chemometric tools, which made it possible to reveal the compounds most impacted by the drying techniques used in this study.

## 2. Materials and Methods

### 2.1. Cocoa Samples

Ripe healthy cocoa pods were harvested by hand during the winter of 2019 in the Germplasm Bank Finca La Represa, belonging to the State Technical University of Quevedo, located in Quevedo (Los Ríos Province, Ecuador). The geographical location is 1°03′18″ south latitude and 79°25′24″ west longitude, at a height of 90 m above sea level.

The cocoa collected belongs to the bulk and fine-flavor genotypes. Bulk cocoa: Forastero (IMC-67 of Amazonian origin) and Trinitarian CCN-51 (Colección Castro Naranjal 51) (ICS-95 (Trinitario) × IMC-67 (Iquitos) × Canelo). Fine-flavor cocoa: ETT103 (National × Venezolano Amarillo type) and La Represa LR14 (Trinitarian × National).

### 2.2. On-Farm Cocoa Processing

Pre drying: The collected cocoa pods were opened, and the beans were spread on a plastic mesh (0.02 m thick) located over a metal structure (0.85 m high, 1 m wide, and 3 m long), to allow the drainage of the mucilaginous material. The cocoa beans were exposed to the Sun for thirteen hours and stirred every three hours in order to obtain a uniform pre drying. The average temperature of the sun drying was 28 °C.

Fermentation: The pre-dried beans were placed inside laurel wood boxes (0.15 × 0.15 × 0.40 m-high cells with 5 kg of capacity and with perforations on the bottom), where the spontaneous fermentation at ambient temperature lasted 3 days. During this period, samples were manually stirred every 24 h with the aim of achieving a better homogeneous fermentation.

Drying: The drying was carried out by applying three techniques; two were natural large-scale techniques and one artificial drying. The first natural technique used was the traditional SD, where the cocoa beans were placed on a concrete floor for 6 days, with 38 h (2 h the first day, 4 h the second day, and 8 h the remaining days) of sun at approximately 31 °C. The second one was a modification of the sun drying, placing the cocoa beans on a black plastic sheeting (SBPD) over a concrete floor for 5 days (the same distribution of drying hours as in the SD), with 30 h of sun at approximately 39 °C. In both experiments, the beans were stirred every three hours, trying to achieve a uniform drying of the beans. At night, the beans were moved to a table located inside a greenhouse, where they were left to ventilate until the next day. The third technique used was a laboratory-scale artificial technique using an oven (OD) (Universal oven UN30, Memmert Gmbh & Co. KG, Schwabach, Germany) for 48 h at 65 °C. The processes concluded when the samples’ humidity (determined using the Ecuadorian Technical standard NTE INEN-ISO 2291:2013) [10] reached 6.5% for all varieties studied.

About 200 g of fresh varietal samples and samples submitted to the three drying processes were placed in plastic bags, which were vacuum sealed and stored at −20 °C to prevent the degradation of the samples until their analysis.

### 2.3. Sample Preparation and HS-SPME Extraction Conditions

Raw or dried cocoa beans were manually deshelled and ground in a Sammic cutter SK-3 food processor (Sevilla, Spain). A total of four grams of ground cocoa samples were weighed and placed in a 10 mL SPME vial. The vials were capped and placed in a Combipal autosampler tray (CTC Analytics, Zwingen, Switzerland). An 80 μm divinylbenzene/carbon wide-range/polydimethylsiloxane (DVB/carbon WR/PDMS) 50/30 mm fiber (Agilent technologies, Basel, Switzerland), previously conditioned according to the supplier’s recommendation, was used for the extraction of the volatiles from cocoa beans via headspace-solid phase microextraction (HS-SPME). The choice of fiber was based on previous cocoa volatile works, where the similar polarity of the different analytes and the polymeric coating allows an extensive identification of the cocoa profile. For the equilibrium step, the vials were incubated at 250 rpm for 5 min at 50 °C, while the extraction was performed at the same equilibrium agitation and temperature conditions and maintaining the agitation for 45 min.

### 2.4. GC-MS Instrumental Parameters

The conditions of the gas chromatography-mass spectrometry (GC-MS) technique were adapted from a previous method developed by Rodríguez-Campos et al. [11]. The desorption time and temperature were set at 10 min and 250 °C, respectively. This process was performed in a Trace GC Ultra gas chromatograph (Thermo Fisher Scientific S.p.A., Rodano, Milan, Italy). Afterwards, the desorbed samples were passed to an ISQ Single MS spectrometer (Thermo Fisher Scientific, Austin, TX, USA). The injection was performed in splitless mode, and an HP-FFAP column of 50 m × 0.32 mm and a 0.50 µm film thickness (SGE Analytical Science, Milton Keynes, U.K.) was used for the separation. The carrier gas was helium at a constant flow rate of 1.7 mL/min. The oven temperature program was set at 40 °C for 5 min, raised to 200 °C at 10 °C/min, and held for 15 min. The MS operated in electron ionization mode at 70 eV using selected-ion-monitoring (SIM) mode. The transfer line and source temperature of the MS were set at 230 °C and 200 °C, respectively. Volatile compounds were tentatively identified as described by Rodríguez Solana et al. [12].

### 2.5. Statistical Analysis

Levene’s and Shapiro–Wilk’s tests were used to, respectively, check the normality and heteroscedasticity of the data, and the variables that failed these parametric assumptions were Box–Cox transformed. Then, the differences in the dataset related to the two factors studied (drying technique and variety) were assessed by means of a two-way ANOVA and a post hoc Tukey’s honestly significant difference (HSD) test. Statistically significant differences were considered at a *p*-value ≤ 0.05. Since the data are given as peak areas with a high range of variation, the results of the ANOVA are expressed as the fold-change to make the interpretation easier. To explore the correlations between metabolites and samples, a hierarchical cluster analysis heatmap was conducted.

This univariate approach enabled studying the variability of the data by taking into account the experimental design. However, it cannot handle the interactions between the volatile metabolites. Therefore, a multivariate approach was performed to study such interactions. First, a principal component analysis (PCA), which is the most-common explorative statistic, was fit on the data. Since this statistic does not allow incorporating information about the experimental design, ANOVA simultaneous component analysis (ASCA), which combines ANOVA and principal component analysis (PCA), was fit on the data. In ASCA, a PCA is built using the effect matrix from ANOVA, obtaining a PCA submodel for each factor, the scores of each PCA being the scores of the factor-level averages. The significance of the factors was calculated by means of a permutation test with N = 1000, since it was large enough to define the tails of the null distribution and to obtain a *p*-value for up to 0.001.

All the statistical analyses were performed using the statistical software R (v. 4.2.2) and the chemometrics tool Solo, running PLS_Toolbox (v. 8.7).

## 3. Results and Discussion

The primary processing of cocoa represents a key point for obtaining cocoa of good quality regarding undesirable aromas and toxic compounds [13,14]. Cocoa drying, the last step prior to cocoa storage and subsequent sale for industrial processing, is one of the oldest techniques used to preserve the food and involves the removal of moisture to provide a safe product for longer periods of time [15]. This step supposes a great challenge for smallholders in order to know how to succeed in obtaining a good-quality cocoa at a good price on the market. The search for economic alternatives, which can be carried out on a large scale allowing the acceleration of the drying process without negatively altering the chemical profile (mainly the degradation of phenolic compounds [16] and the increase in free fatty acids and acetic acid levels [6]) of cocoa, has been a challenge in recent times for farmers. The present work aimed to compare the volatile profile of fine-flavor and bulk cocoa in fresh form and after drying by the traditional SD, its modification using a black plastic sheeting on a concrete floor (SBPD), and a lab-scale OD.

### 3.1. Differences in the Volatile Profile of Dried Cocoa According to the Technique and Genotype

A total of 64 volatiles were identified in the fresh and dried cocoa samples, where 58 were present in samples submitted to the preservation drying process (Supplementary Table S1). These compounds encompass different families, namely alcohols, aldehydes, acids, ketones, esters, terpenes, lactones, and miscellaneous.

**Figure 1:**
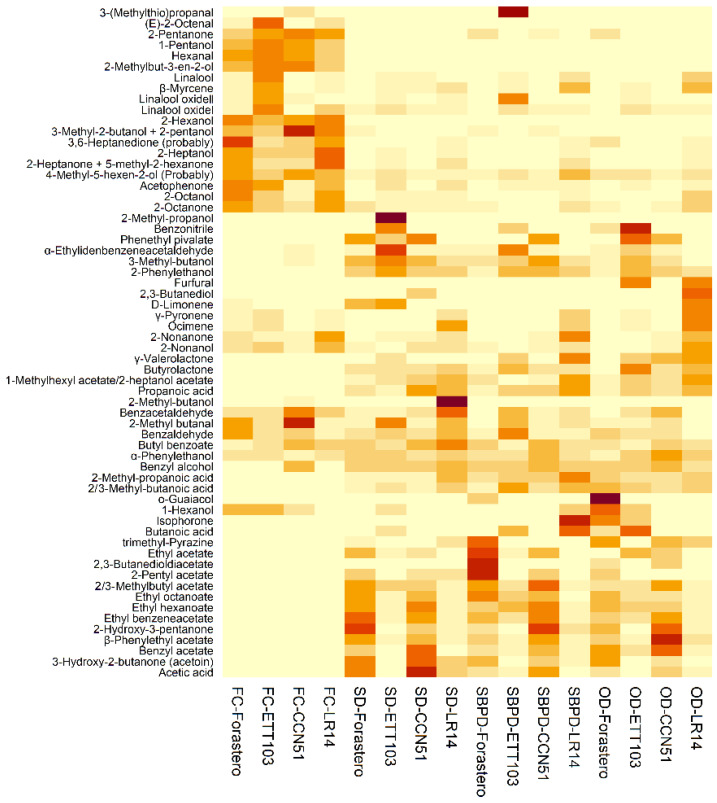
Hierarchical cluster analysis heatmap constructed using normalized areas from volatiles identified in bulk (Forastero and CCN51) and fine-flavor (ETT103 and LR14) cocoa in the fresh state or after drying using the sun drying (SD), sun black plastic sheeting drying (SBPD), and oven drying (OD) techniques.

Alcohols are one of the major groups of compounds found in raw (Figure 1) and fermented cocoa [11], which were preserved to a greater extent using the SD (in bulk and ETT103 cocoa), followed by the OV, and finally, the SBPD (Table 1). Along with ketones, they are the group of compounds less affected by the type of drying with inter-sample variations between 1.12 and 1.18. This family presented the highest amounts (between 1.47- and 2.78-fold) in the samples of the fine-flavor cocoa.

Within this family, the compounds exclusively identified in fresh cocoa were 2-methylbut-3-en-2-ol (fruity and floral notes [17]), 2-hexanol (mushroom and green notes), and 1-pentanol (sweet and pungent aromatic attributes [18]). The reduction in the concentration of alcohols during fermentation was reported as probably caused by volatilization or conversion into other organic compounds due to microbial activity [19]. On the other hand, 1-hexanol, a compound displaying green notes and derived from raw material, was present in some samples of fresh (bulk and ETT-103 varieties) and dried (ETT-103 SD and Forastero-OD and ETT-103 OD cocoa) cocoa, while the undesirable 2,3-butanediol was found in the bulk and ETT-103 SD and LR-14 OD samples.

The primary amyl alcohol 3-methyl-1-butanol, a product of the amino acid catabolism formed during fermentation [20], together with 2-phenylethanol, the most-odor-active compound in fermented and dried cocoa [11], are desirable for high-quality cocoa products, contributing floral and fruity notes [4,21]. Thus, SD and its modification, SBPD, were the drying techniques that better preserved the fermented compounds in both the bulk and fine-flavor cocoa. According to the type of cocoa, ETT-103 was the cultivar with the highest concentrations of both compounds (Table 1).

In the case of the secondary amyl alcohol 3-methyl-2-butanol, this compound is formed in the growing and ripening phase of the fruit pulp and partly infiltrates the seeds, where it remains independent of the fermentation process [21]. Thus, its concentration was higher in the fresh cocoa, and after the drying process, higher amounts were found using controlled drying (SBPD > OD > SD) (Figure 1). Previous authors reported that this compound is developed as a by-product of the biosynthesis or biodegradation of terpenes or fatty acids, indicating a connection with these compounds [21]. Thus, this compound reached its highest concentration in the fine-flavor cocoa varieties, which were richer in terpenoids compared to the bulk cocoa (basic-grade cocoas). In the same way, 2-heptanol (citrus/lemon and fresh notes) and 2-octanol presented higher amounts in the fresh cocoa. The drying techniques that better preserved those compounds were SD and OD. According to Koné et al. [22], 2-heptanol was also found as the main volatile alcoholic compound detected in the raw cocoa, conferring floral and sweet notes to the final cocoa product.

Benzyl alcohol, a compound with sweet and flower attributes, which gradually increases its concentration during the fermentation process [23], was found in higher amounts in the dried cocoa samples. This compound showed higher amounts in the samples submitted to the drying techniques with better control conditions such as OD and SBPD and in the fine-flavor and CCN-51 cocoa genotypes.

High amounts of carbonylic compounds of aldehyde type are crucial for the development of good cocoa flavor and quality [11]. In general, aldehydes can be produced by the oxidation of alcohols derived from short-chain aliphatic aldehydes efficiently produced by the metabolism of yeast [24]. Furthermore, although low concentrations of these compounds may arise even during fermentation and drying, they are usually formed by the Strecker degradation of free amino acids during roasting [25].

**Table 1:** Significance of two-way analysis of variance (ANOVA) for the volatile compounds of cocoa from bulk (Forastero (F) and CCN-51 (C)) and fine-flavor (ETT-103 (E) and LR14 (L)) genotypes and dried by three techniques, namely oven drying (OD), sun drying (SD), and sun drying using a black plastic on a concrete floor (SBPD). Data are expressed as the fold change relative to the maximum peak area of metabolites in each effect.

	Drying Technique				Cocoa Genotype					Drying Technique × Genotype (Interaction)												
	OD	SBPD	SD	*p*	F	C	E	L	*p*	OD × F	SBPD × F	SD × F	OD × C	SBPD × C	SD × C	OD × E	SBPD × E	SD × E	OD × L	SBPD × L	SD × L	*p*
2-Methyl butanal	0.44 b	0.67 b	1.00 a	**	0.14 c	0.29 bc	1.00 a	0.52 b	***	0.14 c	0.06 c	0.09 c	0.35 bc	0.08 c	0.15 c	0.29 bc	0.74 ab	1.00 a	0.04 c	0.37 bc	0.65 ab	***
3-(Methylthio)propanal	0.00 b	1.00 a	0.00 b	***	0.00 b	0.00 b	1.00 a	0.00 b	***	0.00 b	0.00 b	0.00 b	0.00 b	0.00 b	0.00 b	0.00 b	1.00 a	0.00 b	0.00 b	0.00 b	0.00 b	***
Benzaldehyde	0.74	0.93	1.00	ns	0.45 b	0.37 b	1.00 a	0.56 b	***	0.48 bc	0.16 c	0.22 c	0.26 bc	0.16 c	0.27 bc	0.41 bc	1.00 a	0.48 bc	0.08 c	0.25 c	0.72 ab	***
Furfural	1.00 a	0.00 b	0.00 b	***	0.00 c	0.00 c	0.58 b	1.00 a	***	0.00 c	0.00 c	0.00 c	0.00 c	0.00 c	0.00 c	0.58 b	0.00 c	0.00 c	1.00 a	0.00 c	0.00 c	***
Benzacetaldehyde	0.57 b	0.71 b	1.00 a	***	0.14 c	0.61 b	0.76 b	1.00 a	***	0.04 f	0.06 ef	0.10 def	0.50 b	0.12 cdef	0.23 cde	0.27 cd	0.60 b	0.18 cdef	0.07 ef	0.30 c	1.00 a	***
*α*-Ethylidenbenzeneacetaldehyde	0.66 b	0.87 ab	1.00 a	*	0.13 b	0.13 b	1.00 a	0.17 b	***	0.15 c	0.09 c	0.06 c	0.14 c	0.08 c	0.09 c	0.47 b	0.92 a	1.00 a	0.13 c	0.08 c	0.20 bc	**
Aldehydes	0.56 c	0.72 b	1.00 a	***	0.20 c	0.56 b	1.00 a	0.93 a	***	0.11 e	0.08 e	0.12 e	0.50 c	0.13 e	0.24 de	0.33 cd	0.76 b	0.45 c	0.08 e	0.35 cd	1.00 a	***
2-Methyl-propanol	0.00 b	0.00 b	1.00 a	***	0.00 b	0.00 b	1.00 a	0.00 b	***	0.00 b	0.00 b	0.00 b	0.00 b	0.00 b	0.00 b	0.00 b	0.00 b	1.00 a	0.00 b	0.00 b	0.00 b	***
3-Methyl-2-butanol + 2-Pentanol	0.76 b	1.00 a	0.58 c	***	1.00 a	0.77 b	0.74 b	0.44 c	***	0.43 bcd	1.00 a	0.35 cde	0.52 bc	0.56 b	0.30 de	0.53 bc	0.41 bcde	0.37 bcde	0.24 e	0.28 de	0.27 de	***
3-Methyl-butanol	0.50 c	0.78 b	1.00 a	***	0.42 c	0.65 b	1.00 a	0.46 c	***	0.17 g	0.31 efg	0.42 cde	0.25 efg	0.54 bcd	0.60 b	0.57 bc	0.58 bc	1.00 a	0.20 fg	0.43 bcde	0.37 def	***
2-Methyl-butanol	0.00	0.01	1.00	ns	0.01	0.00	0.00	1.00	ns	0.00	0.00	0.00	0.00	0.00	0.00	0.00	0.00	0.00	0.00	0.00	1.00	ns
2-Heptanol	0.83 ab	0.77 b	1.00 a	*	0.11 c	0.22 bc	0.32 b	1.00 a	***	0.07 c	0.12 c	0.11 c	0.15 c	0.22 c	0.23 c	0.23 c	0.12 c	0.54 b	1.00 a	0.90 a	0.89 a	**
4-Methyl-5-hexen-2-ol ^†,††^ (Probably)	1.00 a	0.80 ab	0.54 b	*	0.48 bc	0.32 c	0.74 ab	1.00 a	**	0.67	0.23	0.18	0.36	0.19	0.18	0.55	0.53	0.58	0.86	1.00	0.38	ns
1-Hexanol	1.00 a	0.27 b	0.23 b	***	1.00 a	0.00 d	0.71 b	0.38 c	***	1.00 a	0.00 c	0.00 c	0.00 c	0.00 c	0.00 c	0.39 b	0.00 c	0.32 b	0.00 c	0.38 b	0.00 c	***
2-Octanol	1.00 a	0.37 b	0.84 a	***	0.05 c	0.24 b	0.22 b	1.00 a	***	0.00 d	0.00 d	0.08 d	0.13 cd	0.00 d	0.27 bc	0.00 d	0.10 d	0.25 bc	1.00 a	0.31 b	0.34 b	***
2-Nonanol	1.00 a	0.35 b	0.44 b	***	0.12 c	0.32 b	0.09 c	1.00 a	***	0.07 de	0.06 de	0.05 de	0.13 cd	0.13 cd	0.21 bc	0.06 de	0.00 e	0.07 de	1.00 a	0.25 b	0.22 bc	***
2.3-Butanediol	1.00 a	0.00 b	0.28 b	***	0.02 b	0.25 b	0.01 b	1.00 a	***	0.00 b	0.00 b	0.02 b	0.00 b	0.00 b	0.25 b	0.00 b	0.00 b	0.01 b	1.00 a	0.00 b	0.00 b	***
*α*-Phenylethanol	1.00 a	0.72 b	0.73 b	***	0.56 c	0.88 b	0.88 b	1.00 a	***	0.36 f	0.43 ef	0.47 def	0.93 a	0.63 bcd	0.41 ef	0.74 b	0.57 bcde	0.65 bc	1.00 a	0.54 cde	0.69 bc	***
Benzyl Alcohol	1.00 a	0.95 a	0.82 b	**	0.85 b	0.87 ab	0.88 ab	1.00 a	*	0.94 a	0.86 a	0.51 b	0.85 a	0.79 ab	0.72 ab	0.74 ab	0.93 a	0.73 ab	1.00 a	0.76 ab	0.95 a	**
2-Phenylethanol	0.84 b	0.89 b	1.00 a	***	0.32 d	0.62 c	1.00 a	0.74 b	***	0.28 g	0.19 g	0.42 f	0.57 de	0.52 ef	0.62 cde	0.76 b	0.97 a	1.00 a	0.65 bcd	0.71 bc	0.65 bcd	***
Alcohols	0.89 b	0.85 c	1.00 a	***	0.36 c	0.67 b	1.00 a	0.99 a	***	0.26 f	0.23 f	0.40 e	0.50 d	0.51 d	0.66 c	0.69 c	0.83 b	0.98 a	1.00 a	0.75 bc	0.71 c	***
Acetic acid	0.47 b	0.47 b	1.00 a	***	0.59 b	1.00 a	0.12 c	0.43 b	***	0.29 bcd	0.28 bcd	0.50 b	0.35 bcd	0.45 bc	1.00 a	0.09 cd	0.09 cd	0.04 d	0.20 bcd	0.11 cd	0.45 bc	**
Propanoic acid	1.00	0.92	0.95	ns	0.21 c	0.58 b	0.52 b	1.00 a	***	0.14 f	0.11 f	0.29 ef	0.39 cdef	0.36 def	0.78 abc	0.57 bcde	0.56 bcde	0.23 ef	1.00 a	0.90 ab	0.71 abcd	**
2-Methyl-propanoic acid	0.96 a	1.00 a	0.55 b	***	0.36 c	0.39 bc	0.50 b	1.00 a	***	0.51 cd	0.27 ef	0.10 f	0.34 de	0.38 de	0.23 ef	0.41 cde	0.55 cd	0.25 ef	0.83 ab	1.00 a	0.62 bc	**
Butanoic acid	0.74 b	1.00 a	0.19 c	***	0.17 c	0.00 d	1.00 a	0.55 b	***	0.31 c	0.00 d	0.00 d	0.00 d	0.00 d	0.00 d	0.89 a	0.63 b	0.30 c	0.00 d	1.00 a	0.00 d	***
2-/3-Methyl-butanoic acid	1.00 a	0.85 a	0.53 b	***	0.50 b	0.37 b	0.89 a	1.00 a	***	0.85 ab	0.20 d	0.15 d	0.41 cd	0.29 d	0.19 d	0.66 abc	1.00 a	0.49 cd	0.88 ab	0.88 ab	0.65 bc	***
Acids	0.58 b	0.56 b	1.00 a	***	0.63 b	1.00 a	0.24 c	0.56 b	***	0.37 bcd	0.30 bcd	0.51 bc	0.39 bcd	0.48 bc	1.00 a	0.16 cd	0.20 bcd	0.09 d	0.31 bcd	0.22 bcd	0.52 b	**
2-Pentanone	0.94 a	1.00 a	0.00 b	***	1.00 a	0.28 b	0.00 c	0.00 c	***	0.92 a	1.00 a	0.00 c	0.27 b	0.27 b	0.00 c	0.00 c	0.00 c	0.00 c	0.00 c	0.00 c	0.00 c	***
2-Heptanone + 5-methyl-2-hexanone	0.87	0.79	1.00	ns	0.15 c	0.24 bc	0.37 b	1.00 a	***	0.18	0.12	0.10	0.16	0.22	0.28	0.45	0.11	0.46	0.79	0.99	1.00	ns
2-Octanone	1.00 a	0.52 b	0.69 b	**	0.54 b	0.21 c	0.28 c	1.00 a	***	0.36 bc	0,36 bc	0,27 bcd	0.20 bcd	0,00 d	0,19 bcd	0.12 bcd	0,05 cd	0,34 bc	1.00 a	0,46 b	0,36 bc	***
3-Hydroxy-2-butanone (acetoin)	0.56 b	0.60 b	1.00 a	***	1.00 a	0.73 b	0.05 d	0.34 c	***	0.80 ab	0.72 abc	0.66 abc	0.23 de	0.36 cde	1.00 a	0.03 e	0.05 e	0.04 e	0.14 de	0.15 de	0.45 bcd	***
2-Hydroxy-3-pentanone	1.00	0.85	0.82	ns	0.81 b	1.00 a	0.00 d	0.41 c	***	0.67 ab	0.42 bc	0.92 a	1.00 a	0.96 a	0.51 bc	0.00 d	0.00 d	0.00 d	0.40 bc	0.36 bc	0.26 cd	***
2-Nonanone	1.00	0.86	0.64	ns	0.06 b	0.20 b	0.12 b	1.00 a	***	0.03	0.05	0.04	0.13	0.11	0.19	0.05	0.01	0.22	1.00	0.87	0.32	ns
3,6-Heptanedione ^‡^ (probably)	1.00 a	0.32 b	0.25 b	***	0.56 b	0.00 c	0.00 c	1.00 a	***	1.00 a	0.00 d	0.05 d	0.00 d	0.00 d	0.00 d	0.00 d	0.00 d	0.00 d	0.86 a	0.60 b	0.41 c	***
Acetophenone	0.78 ab	0.77 b	1.00 a	*	0.25 b	0.32 b	0.84 a	1.00 a	***	0.25 d	0.19 d	0.20 d	0.30 cd	0.23 d	0.28 d	0.46 bcd	0.74 ab	0.92 a	0.86 a	0.68 abc	1.00 a	*
Ketones	0.98	0.89	1.00	ns	0.34 b	0.35 b	0.27 b	1.00 a	***	0.39 bc	0.33 bc	0.24 bc	0.24 bc	0.28 bc	0.48 b	0.28 bc	0.10 c	0.39 bc	0.98 a	1.00 a	0.82 a	**
Ethyl Acetate	0.58 b	1.00 a	0.48 b	***	1.00 a	0.70 b	0.55 c	0.22 d	***	0.00 e	1.00 a	0.27 c	0.31 c	0.32 bc	0.26 cd	0.46 b	0.12 e	0.12 de	0.12 e	0.09 e	0.07 e	***
2-Pentyl acetate	0.25 c	1.00 a	0.45 b	***	1.00 a	0.24 b	0.00 d	0.13 c	***	0.30 b	1.00 a	0.19 c	0.00 d	0.21 c	0.15 c	0.00 d	0.00 d	0.00 d	0.00 d	0.00 d	0.20 c	***
3-Methylbutyl acetate/ 2-methylbutyl acetate	0.69 b	1.00 a	0.68 b	***	0.97 a	1.00 a	0.63 b	0.40 c	***	0.32 def	1.00 a	0.49 c	0.68 b	0.77 b	0.44 cd	0.40 cde	0.37 cdef	0.42 cd	0.24 f	0.25 f	0.26 ef	***
Ethyl hexanoate	0.89 ab	1.00 a	0.81 b	*	0.91 a	1.00 a	0.71 b	0.32 c	***	0.77 ab	0.74 ab	0.60 bc	0.55 bcd	0.77 ab	1.00 a	0.60 bc	0.75 ab	0.29 cde	0.32 cde	0.26 de	0.16 e	***
1-Methylhexyl acetate/ 2-heptanol acetate	1.00 a	0.63 b	0.71 b	**	0.23 b	0.33 b	0.34 b	1.00 a	***	0.11 ef	0.26 cdef	0.13 ef	0.19 cdef	0.15 def	0.37 bcde	0.45 bcd	0.05 f	0.24 cdef	1.00 a	0.64 b	0.50 bc	***
Ethyl octanoate	0.77 b	1.00 a	0.67 b	***	1.00 a	0.59 b	0.44 c	0.20 d	***	0.59 b	1.00 a	0.44 cd	0.28 ef	0.36 de	0.55 bc	0.36 de	0.38 de	0.14 fg	0.19 fg	0.11 g	0.11 g	***
2,3-Butanedioldiacetate	0.50 b	1.00 a	n.d.	***	1.00 a	0.20 b	n.d.	0.05 c	***	0.20 b	1.00 a	0.00 c	0.24 b	n.d.	0.00 c	n.d.	n.d.	0.00 c	0.06 c	n.d.	0.00 c	***
Benzyl acetate	1.00 a	0.43 c	0.57 b	***	0.61 b	1.00 a	0.25 c	0.31 c	***	0.81 a	0.28 bc	0.28 bc	0.81 a	0.42 b	1.00 a	0.40 b	0.16 cd	0.00 d	0.40 b	0.18 cd	0.10 cd	***
Ethyl benzeneacetate	0.92	1.00	1.00	ns	0.95 a	1.00 a	0.46 b	0.42 b	***	0.59 bcd	0.93 ab	1.00 a	0.82 abc	0.87 ab	0.97 a	0.50 cd	0.45 d	0.27 d	0.46 d	0.32 d	0.33 d	**
*β*-Phenylethyl acetate	1.00 a	0.64 b	0.68 b	***	0.68 b	1.00 a	0.29 d	0.45 c	***	0.44 b	0.49 b	0.49 b	1.00 a	0.50 b	0.59 b	0.24 c	0.17 c	0.19 c	0.50 b	0.22 c	0.22 c	***
Phenethyl pivalate	0.83 b	0.29 c	1.00 a	***	0.29 c	1.00 a	0.83 b	0.00 d	***	0.00 d	0.00 d	0.49 c	0.42 c	0.50 c	0.80 b	1.00 a	0.00 d	0.43 c	0.00 d	0.00 d	0.00 d	***
Butyl benzoate	0.60 b	0.69 b	1.00 a	***	0.58 b	0.78 b	0.33 c	1.00 a	***	0.28 cd	0.48 bc	0.31 bcd	0.38 bcd	0.48 bc	0.58 b	0.20 cd	0.14 d	0.27 cd	0.44 bc	0.39 bcd	1.00 a	***
Esters	0.96 a	1.00 a	0.77 b	***	0.93 a	1.00 a	0.47 b	0.44 b	***	0.39 efg	1.00 a	0.54 cd	0.85 b	0.65 c	0.59 c	0.42 de	0.27 gh	0.29 fgh	0.41 ef	0.25 h	0.26 h	***
*β*-Myrcene	1.00	0.65	0.55	ns	0.01 b	0.10 b	0.18 b	1.00 a	***	0.01	0.00	0.01	0.05	0.02	0.14	0.16	0.09	0.12	1.00	0.68	0.40	ns
D-Limonene	1.00 a	0.00 b	0.76 a	***	0.23 bc	0.00 c	0.47 b	1.00 a	***	0.00 c	0.00 c	0.24 bc	0.00 c	0.00 c	0.00 c	0.00 c	0.00 c	0.49 b	1.00 a	0.00 c	0.04 c	***
Ocimene (Isomers E and Z)	1.00 a	0.38 b	0.48 b	**	0.00 b	0.01 b	0.13 b	1.00 a	***	0.00 d	0.00 d	0.00 d	0.02 d	0.00 d	0.00 d	0.15 cd	0.05 cd	0.04 cd	1.00 a	0.39 bc	0.52 b	**
*γ*-Pyronene	1.00 a	0.33 b	0.23 b	***	0.00 b	0.00 b	0.10 b	1.00 a	***	0.00 c	0.00 c	0.00 c	0.00 c	0.00 c	0.00 c	0.13 bc	0.00 c	0.04 c	1.00 a	0.37 b	0.23 bc	***
Linalool oxide I	1.00 a	0.78 b	0.89 ab	*	0.18 d	0.45 c	1.00 a	0.67 b	***	0.12 ef	0.00 f	0.35 de	0.47 cd	0.37 de	0.37 de	1.00 a	0.89 a	0.81 ab	0.72 abc	0.55 bcd	0.53 bcd	*
Linalool oxide II	0.36	1.00	0.39	ns	0.13 b	0.14 b	1.00 a	0.29 b	**	0.08 b	0.05 b	0.04 b	0.04 b	0.05 b	0.09 b	0.17 b	1.00 a	0.17 b	0.14 b	0.09 b	0.16 b	*
Linalool	1.00	0.61	0.59	ns	0.05 b	0.24 b	0.37 b	1.00 a	***	0.03 b	0.03 b	0.01 b	0.04 b	0.02 b	0.34 b	0.17 b	0.17 b	0.26 b	1.00 a	0.54 ab	0.12 b	**
Terpenes	1.00	0.67	0.58	ns	0.05 b	0.16 b	0.38 b	1.00 a	***	0.03 c	0.02 c	0.03 c	0.05 c	0.03 c	0.21 bc	0.19 bc	0.28 bc	0.22 bc	1.00 a	0.53 ab	0.28 bc	**
Valerolactone	1.00 a	0.65 b	0.14 c	***	0.00 d	0.25 c	0.68 b	1.00 a	***	0.00 d	0.00 d	0.00 d	0.45 bc	0.00 d	0.00 d	0.50 b	0.46 bc	0.27 c	1.00 a	0.80 a	0.00 d	***
Butyrolactone	1.00 a	0.64 b	0.57 b	***	0.16 c	0.41 b	1.00 a	0.96 a	***	0.05 e	0.09 de	0.17 cde	0.26 cde	0.21 cde	0.31 cd	0.88 ab	0.68 b	0.35 c	1.00 a	0.42 c	0.41 c	***
Lactones	1.00 a	0.64 b	0.52 b	***	0.15 c	0.41 b	1.00 a	1.00 a	***	0.04 g	0.08 fg	0.15 efg	0.29 def	0.18 efg	0.27 defg	0.83 ab	0.65 bc	0.34 de	1.00 a	0.46 cd	0.36 de	***
trimethyl-Pyrazine	1.00 a	0.66 b	0.22 c	***	1.00 a	0.25 c	0.05 c	0.54 b	***	0.62 b	1.00 a	0.08 d	0.42 bc	0.00 d	0.00 d	0.09 d	0.00 d	0.00 d	0.54 bc	0.10 d	0.29 cd	***
Isophorone	1.00 a	0.85 a	0.00 b	***	0.77 b	0.00 d	0.41 c	1.00 a	***	0.77 b	0.00 d	0.00 d	0.00 d	0.00 d	0.00 d	0.41 c	0.00 d	0.00 d	0.00 d	1.00 a	0.00 d	***
Benzonitrile	1.00 a	0.26 b	0.49 b	***	0.15 b	0.00 b	1.00 a	0.00 b	***	0.29 bc	0.00 c	0.00 c	0.00 c	0.00 c	0.00 c	1.00 a	0.34 bc	0.64 ab	0.00 c	0.00 c	0.00 c	**
*o*-Guaiacol	1.00 a	0.29 b	0.00 b	***	1.00 a	0.00 b	0.00 b	0.00 b	***	1.00 a	0.29 b	0.00 b	0.00 b	0.00 b	0.00 b	0.00 b	0.00 b	0.00 b	0.00 b	0.00 b	0.00 b	***
Miscellaneous	1.00 a	0.62 b	0.13 c	***	1.00 a	0.09 d	0.42 c	0.61 b	***	1.00 a	0.39 c	0.02 fg	0.13 de	0.00 g	0.00 g	0.44 c	0.06 efg	0.11 de	0.16 d	0.62 b	0.09 def	***
Total	0.65 b	0.63 b	1.00 a	***	0.64 b	1.00 a	0.40 c	0.72 b	***	0.39 bc	0.34 bc	0.52 bc	0.46 bc	0.51 bc	0.35 bc	0.26 c	0.31 c	0.22 c	0.44 bc	0.35 bc	0.63 b	**

Compounds tentatively identified according to the linear retention index (RI) calculated in previous works: 4-methyl-5-hexen-2-ol, ^†^ Costa Castro Alves et al. [26] and ^††^ Li [27]; 3,6-heptanedione, ^‡^ Raffo et al. [28]. RI: retention index; Ion Q: ion of quantification. In the same raw, different letters indicate significant differences among the different drying techniques, cultivars or drying techniques × cultivar interactions (*p* < 0.05) according to post hoc Tukey HSD tests. Ns: not significant; *p*-values lower than 0.05, 0.01, and 0.001 are designated with one, two, or three asterisks (*).

Hexanal, with green notes, and (E)-2-octenal, with undesirable fatty and waxy aroma [29], are potent odorants that arise from lipid precursors [30]. As was previously published [31], both aldehydes are characteristic of fresh cocoa and are rarely found in dried cocoa (Figure 1). According to the drying process, the SD-type techniques produced higher amounts of aldehydes. The traditional SD highlighted the content of compounds that result from the Strecker degradation of free amino acids [21], the aldehydes 2-methyl butanal (malty, chocolate, and cocoa aromas) [25] in fine-flavor cocoa samples and benzacetaldehyde (green notes, honey, floral rose, sweet, powdery, fermented, chocolate with a slight earthy nuance) [32] in the LR14 cocoa samples. The SBPD treatment (modification of SD) produced higher amounts of the unpleasant 3-(methylthio)propanal (methional; with potato, foul, sulfurous, onion, vegetable, and persistent notes [31]), in the variety ETT103 cocoa. Moreover, in this varietal sample, it is worth noticing the high content of *α*-ethylidenbenzeneacetaldehyde (musty, sweet narcissus cortex, beany honey, cocoa, nutty radish aromatic characteristics) using both sun drying techniques.

Furfural, a compound resulting from the thermal treatment during the processing of cocoa and related to the breakdown of carbohydrates, with a sweet-caramel-like aroma [33], was only identified in the fine-flavor cocoa submitted to OD, probably due to the higher temperatures reached for a longer time with this technique and the susceptibility of this type of cocoa to carbohydrate degradation.

Besides aldehydes, high amounts of ketones are desirable to obtain cocoa with a high aromatic quality [11]. Acetophenone, a compound with a low-odor-active value (OAV) and characterized by enhancing the floral character of fine-flavor varieties [19], showed the highest content in the fresh cocoa from Forastero and the fine-flavor varieties. Furthermore, the highest amounts found in dried cocoa were in the ETT-103 and LR-14 samples using mainly the sun drying techniques. On the other hand, acetoin with buttery notes is an important and desirable compound for a good cocoa fermentation, and its relevance also lies in being considered a precursor of tetramethylpyrazine (an important odor-active component of cocoa flavor) [25]. This compound could be produced by alcohol fermentation from pyruvate and butanediol [31], justifying in this way its absence in fresh cocoa, and presenting higher mounts in the SD CCN-51 and Forastero samples dried by all techniques.

Esters (ethyl and methyl esters and acetates) are considered the second-most-important class of volatiles in cocoa after pyrazines [4,34]. They confer a fruity flavor and are the typical aroma components (mainly acetates) in unroasted cocoas that arise from amino acids [20]. Although high temperatures during roasting negatively affect the content of these compounds [34], in the case of the dried samples submitted to mild temperatures, the content increased considerably in relation to the fresh cocoa in the more controlled drying techniques, OD and SBPD (Figure 1).

According to Figure 1, the presence of acetates in the dried samples is an important difference compared to the fresh ones (absence). Adler et al. [35] postulated that the formation of acetates is a key step in the development of desirable flavors. These compounds are formed from alcohols that are partly esterified by acetic acid to form acetates. They are produced during the last days of fermentation when the alcohols are enzymatically esterified by acetic acid, which is generated within this step [21].

Ethyl acetate, with pineapple notes, is a product of esterification from acetic acid and ethanol [4]. We observed that dried cocoa presented a variable response in the ethyl acetate content depending on the drying technique used. CCN-51 and LR14, both trinitarian mixed cocoa, presented similar amounts regardless of the drying technique, while in Forastero, the highest content was obtained with SBPD, while in ETT-103 when OD was used. The compound 3-methylbutyl acetate, probably produced from 3-methyl-1-butanol oxidation, increased during the drying process. Its highest amounts were found in the bulk cocoa, using mainly the SBPD (Forastero and CCN51) and OD (CCN51) techniques, while in the dried fine-flavor cocoa, was found in similar concentrations regardless of the drying technique used.

*β*-Phenylethyl acetate (2-phenylethylacetate), a compound from the metabolism of yeasts and with flowery and honey notes, was defined as the principal compound responsible for the characteristic aroma of Asian cocoa liquor [36]. The highest content was found in trinitarian-type cocoa (CCN-51 and LR14) and using artificial drying (OD), while similar amounts were found in Forastero and ETT103 regardless of the drying technique used. High levels of this compound and low concentrations of the amyl acetate 3-methyl-1-butanol acetate are important to obtain high aromatic quality cocoa [11]. In general, the formation of amyl acetates must be avoided during the fermentation step, since they are considered as indicators of flavor defects.

Terpenes are considered potential key floral components of fine-flavor cocoa [19]. In particular, linalool, *β*-myrcene, and ocimene may be responsible for the fine flavor, contributing to floral/tea-like, spicy, and floral/herbal notes, respectively. These compounds derive via the methyl-erythritol 4-phosphate pathway in the plastids, with carbohydrates as precursors. The three terpenes already mentioned were more abundant in fine-flavor varieties. ETT-103 cocoa samples showed the highest content in fresh samples; however, after drying, LR-14 retained them to a higher extent mainly when using OD. In the case of linalool oxide isomers I and II, the highest amounts were found in fresh cocoa samples and especially in the fine-flavor varieties. After drying, ETT-103 submitted to OD (for the isomer II) or any of the drying techniques used (in the case of isomer I) showed the highest amounts.

Short-chain carboxylic acids (acetic acid, 2-methylpropanoic acid, or isobutyric, 3-methylbutanoic acid, or isovaleric and propionic acid), with rancid, butter, and hammy off-odor notes, are compounds that predominate in fermented cocoa beans and increase during prolonged fermentation as a result of sugar metabolism [25]. However, we can find in the literature that their concentration could be reduced [37] or increased [4] during drying, and it is demonstrated that, in some cases, these compounds can be diminished or even eliminated during the roasting and conching stages, which is the case of acetic acid at 70% [11].

By the nature of its origin, it is reasonable that acidic compounds in the fresh samples were absent, while in the dried cocoa, the bulk (mainly CCN51) and the trinitarian fine-flavor cocoa LR14 showed the highest amounts.

Acetic acid, a compound with a sour and vinegar-like aroma and considered the highest odor-active compound in fermented and unroasted beans, is produced in the pulp during fermentation and causes the acid and sour taste of raw cocoa [20]. The acidification of cocoa beans by acetic acid during fermentation leads to various biochemical modifications necessary for cocoa flavor development. This compound, together with propionic acid, did not show significant differences among the drying techniques used, except for the CCN51 variety, which was more affected (higher amounts) using the SD technique. In this case, the use of a higher speed and temperature during drying (OD and SBPD) in comparison to the traditional SD allowed the content of these compounds to be reduced to less than half.

For the rest of the acidic compounds, in general, it seems that the OD (Forastero) or OD and SBPD (fine-flavor cocoa) techniques produced higher amounts. However, these compounds have less impact on the final cocoa off-flavor.

Pyrazines are an important family of volatiles and key odor compounds in cocoa aroma. Among these compounds, tetramethylpyrazine and trimethylpyrazine are the most-important, displaying nutty, grassy, and persistent cocoa notes [38]. Pyrazines such as tetramethylpyrazine and trimethylpyrazine can be formed during the fermentation of cocoa beans as a metabolic product of *Bacillus subtilis* and *B. megatrium*, respectively. However, when endogenous yeasts are used, the roasted cocoa beans present less and have a less chocolaty flavor [25]. Most of these compounds originate from *α*-aminoketones by Strecker degradation and Maillard reactions during the drying (due to a decrease in humidity and temperatures between 30 °C and 50 °C) and roasting processes [10]. In our study, the only pyrazine identified was trimethylpyrazine in the Forastero and LR-14 cocoa samples obtained using all the drying techniques, while in CCN51 and ETT103, only for the samples obtained with the OD technique. Samples submitted to the OD (CCN51, ETT103 and LR14) and especially SBPD (Forastero) techniques presented the highest amounts. The lowest amounts found in fine-flavor cocoa were in accordance with the low concentrations found in national (Arriba) cocoa [20,39].

### 3.2. Chemometric Evaluation of Results

#### 3.2.1. Combined Effect of Drying Technique and Variety on Cocoa Volatiles

**Figure 2:**
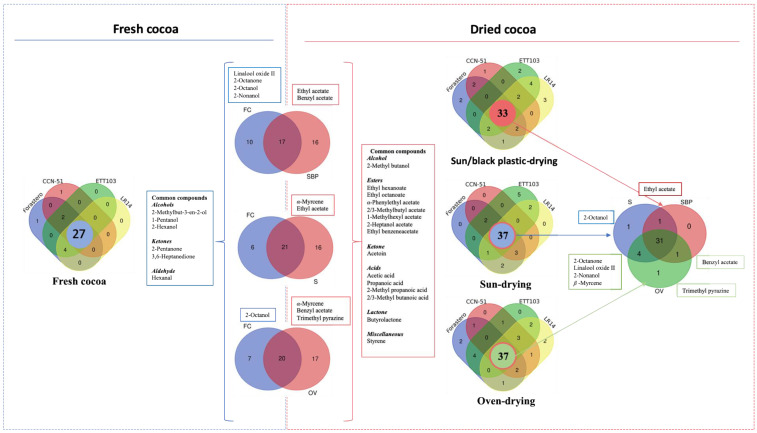
Venn diagrams of volatile compounds from bulk (Forastero and CCN51) and fine-flavor (ETT103 and LR14) cocoa samples in fresh form and after drying by oven, sun, and a modification of sun drying using a black plastic on a concrete floor.

**Figure 3:**
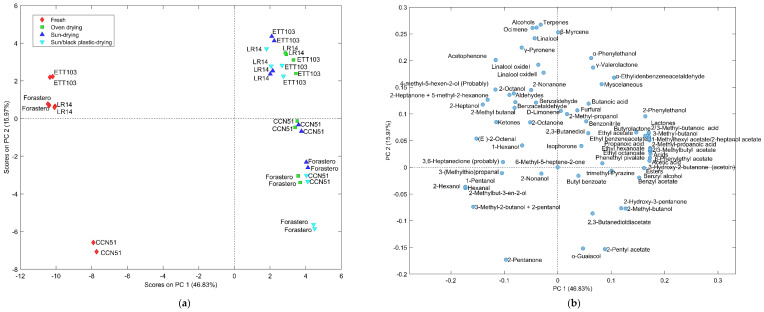
Scores (**a**) and loadings (**b**) of principal component analysis (PCA) constructed with volatiles from fresh and dried bulk (Forastero and CCN-51) and fine-flavor (ETT-103 and LR-14) cocoa.

**Figure 4:**
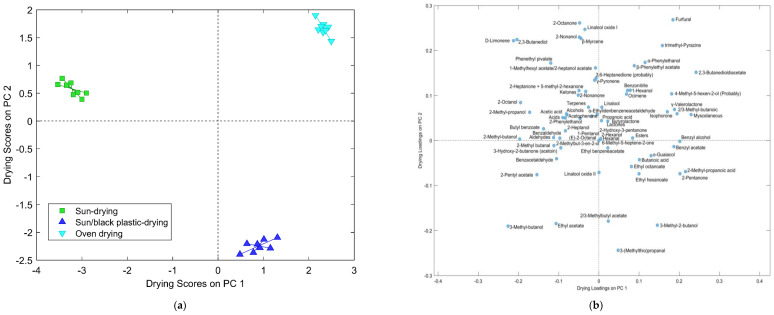
Scores (**a**) and loadings (**b**) plot on Principal Components 1 and 2 of the ASCA performed to evaluate the main effect of drying using the volatile content of cocoa submitted to sun drying, a modification using a black plastic on a concrete floor, and oven drying.

In order to compare the volatile content between fresh and dried cocoa and to evaluate the influence of the drying technique used according to the variety studied, Figure 2, Figure 3 and Figure 4 show the results analyzed using Venn diagrams, principal component analysis (PCA), and ANOVA simultaneous component analysis (ASCA).

As expected, clear differences can be observed between fresh and dried samples in the Venn diagrams (Figure 2). Since the fresh cocoa was not submitted to primary processing, where different precursors of flavor and aroma are developed, those samples presented a lower number of volatiles (28 compounds) identified in relation to the dried cocoa. Thus, the fresh cocoa is distinguishable from the dry one by its content of the alcohols 2-methylbut-3-en-2-ol, 1-pentanol, and 2-hexanol, the ketones 2-pentanone and 3,6-heptanedione, and the aldehyde hexanal. In the case of the dried samples, 37 compounds were identified in the OD and SD cocoa and 33 compounds using the SBPD technique. The higher number of common compounds using the OD and SD techniques indicates higher drying homogenization in the different bulk and fine-flavor cocoa. Furthermore, both techniques had the highest number of compounds matching the fresh cocoa, indicating a higher preservation potential. In the case of the dried samples, differentiation of the compounds is represented by the alcohol 2-methyl butanol, esters such as ethyl hexanoate and ethyl octanoate, the acetates *α*-phenylethyl acetate, 2 or 3-methylbutyl acetate, 1-methylhexyl acetate, 2-heptanol acetate, and ethyl benzeneacetate, the ketone acetoin, the acids acetic, propanoic, 2-methyl propanoic, and 2 or 3-methyl butanoic, and butyrolactone. These compounds are produced during the fermentation and/or drying steps. Comparing the drying techniques, 31 compounds were common to all studied techniques. Moreover, the samples obtained with the SD and OD techniques showed higher amounts in the terpenes linalool oxide II and *β*-myrcene, the alcohol 2-nonanol, and the ketone, 2-octanone. The SD cocoa samples also presented 2-octanol, a compound with a negligible contribution to the overall aroma due to its low OAVs [19], while OD was the only technique preserving trimethyl pyrazine in all samples, indicating a good fermentation process and predicting the high quality of the final samples. Finally, SBPD presented higher acetate content, ethyl acetate (fruity notes), and benzyl acetate, compounds common to the SD and OD techniques, respectively.

To complete this study, a comparison of the volatile profile among fresh cocoa samples and those dried by the SD, SBPD, and OD techniques was carried out with the aid of principal component analysis (PCA) (Figure 3). Components **1** (PC1) and **2** (PC2) accounted for 46.83% and 15.97%, respectively, of the total variance. PC1 allowed the grouping of samples by type of processing (fresh and dried cocoa), fresh and dried cocoa being positioned in the negative and positive scores values, respectively. Meanwhile, PC2 showed a clear separation of the dried samples according to the type of variety studied. As can be observed in the loadings plot, the fresh cocoa presented a higher content of alcohols (2-methylbut-3-en-2-ol, 1-pentanol, 3-methyl-2-butanol + 2-pentanol, 2-hexanol, 4-methyl-5-hexen-2-ol, 2-heptanol, and 2-octanol), aldehydes (2-methyl butanal, hexanal, (E)-2-octenal and 3-(methylthio)propanal), and ketones (2-pentanone, 2-heptanone or 5-methyl-2-hexanone, 3,6-heptanedione, and acetophenone), compounds characteristic of this type of sample [7]. In relation to the drying technique, it can be observed that the fine-flavor varieties (ETT103 and LR14) did not present a clear difference in terms of their volatile composition among the different drying techniques studied. As was expected for this type of cocoa, fine-flavor cocoa showed higher amounts of terpenes, the fresh samples being more abundant in linalool and its derivatives (linalool oxide isomers I and II), while dried samples were characterized by higher amounts in ocimene, d-limonene, and *γ*-pyronene, as well as the compounds *α*-phenylethanol, valerolactone, and *α*-ethylidenbenzeneacetaldehyde.

On the other hand, the OD and SD bulk cocoa samples showed a volatile profile with higher similarities and differentiated from the samples dried using the SBPD technique characterized by a higher content of esters (2/3-methylbutyl acetate, ethyl benzeneacetate, ethyl hexanoate, 1-methylhexyl acetate or 2-heptanol acetate, *β*-phenylethyl acetate, phenylethyl pivalate, and ethyl octanoate) and acids (acetic acid, propanoic acid, 2-methyl propanoic acid, and 2/3-methyl butanoic acid).

#### 3.2.2. Effects of Drying Technique and Variety

ANOVA simultaneous component analysis (ASCA) was applied to find differences in the cocoa volatile profile and to extract useful information and a better understanding on the effects of each factor, the drying technique used, and the variety studied, as well as their interactions. The significance of the factors and of their interaction effects were assessed by means of a permutation test where the results (explained variance or effect, *p*-value, and cumulative variance by component) are shown in Table 2. Both studied factors and their interaction were statistically significant (*p* < 0.001). It was observed that the variety factor explained the highest variance (55.02% of the total) in the cocoa samples related to the volatile profile. The drying effect showed a lower effect (13.21%), while its interaction with the variety explained a high 29.39% of the total variance. This means that the variety type is the factor with most influences the volatile profile, while the technique of drying, although having a lower effect, can have an important contribution to the final aroma of the cocoa (main effects). Thus, the changes in the profile are noteworthy when different drying processes were performed on the different varieties assessed (interactions). Therefore, these results reveal the need to optimize the drying process in each variety to obtain cocoa with improved quality, as was addressed in this study. In order to interpret separately the observed variation related to each effect, a plot with the corresponding principal component analysis of ASCA is given. 

Scores and loading plots of Components **1** and **2** (explaining 100% of the variance) of the main effect, the drying technique, are shown in Figure 4a,b, respectively. From the scores plot, it is possible to observe a clear separation of the drying techniques distributed in the first (OD), second (SD), and fourth (SBPD) quadrants, where the positive PC1 grouped the techniques that reached higher drying temperatures (OD and SBPD). The OD technique tended to produce higher amounts of furfural and trimethyl-pyrazine, compounds characteristic of applying a thermal treatment to the samples, the alcohols 4-methyl-5-hexen-2-ol, 2-nonanol, and benzyl alcohol, esters such as 2,3-butanedioldiacetate and benzyl acetate, terpenes such as linalool oxide II and *β*-myrcene, acids 2/3-methyl butanoic and 2-methylpropanoic, and valerolactone, and isophorone. In the case of cocoa samples dried using the SD technique, they tended to present higher levels of the alcohols 2-methylpropanol, 2-methylbutanol, 3-methyl-butanol, 2,3-butanediol, and 2-octanol. Finally, the cocoa samples obtained following the SBPD technique were characterized by higher amounts mainly of the aldehyde 3-(methylthio)propanal, the alcohol 3-methyl-2-butanol or 2-pentanol, and the esters ethyl acetate and 2/3-methylbutyl acetate.

## 4. Conclusions

On the basis of the results obtained, we can conclude that the drying process exerted profound effects on the volatile profile of cocoa. A comparison between the fresh and dried samples indicated that the fresh cocoa, regardless of the variety, was defined by its higher content of certain alcohols (1-pentanol, 2-methylbut-3-en-2-ol, 2-hexanol, 3-methyl-2-butanol + 2-pentanol, 2-heptanol, 4-methyl-5-hexen-2-ol, and 2-octanol), aldehydes ((E)-2-octenal and hexanal), and ketones (2-pentanone, 3,6-heptanedione, 2-heptanone + 5-methyl-2-hexanone, acetophenone, and 2-octanone), while the dried samples stood out for their amounts of esters (mainly acetates), acids, and butyrolactones. In addition, the chemometric evaluation of the volatiles indicated variations in the aromatic quality of the cocoa, rather marked by the variety of cocoa studied, but also influenced by its interaction with the drying technique used. The volatile content of the fine-flavor varieties was slightly affected by the drying technique used. In the case of the bulk varieties, samples obtained following the OD and SD techniques showed similar volatile profiles, while the samples obtained following the SBPD technique presented a more differentiated profile characterized by lower acid content and good preservation of beneficial compounds such as certain alcohols (e.g., 2-phenylethanol) and esters (e.g., ethyl acetate). Therefore, our findings demonstrated that the modified conventional sun drying using a black plastic sheeting can be effectively used by cocoa producers (smallholders) to produce cocoa of quality considering its drying efficiency and low production costs and drying times (increased radiation absorption and heat transmittance into the cocoa samples) in an accessible way, in comparison to the convectional SD and to the controlled lower-scale OD techniques. However, more in-depth studies should be performed to confirm its suitability considering other important quality parameters such as bioactive compounds and unfavorable compounds such as ochratoxin A, biogenic amines, etc.

## Figures and Tables

**Table 2:** ANOVA simultaneous component analysis (ASCA) to evaluate the effect of the main factors, drying and variety, and their interaction on the volatile content of cocoa.

Term	Effect (%)	*p*-Value	% Cumulative Variance by Component
Drying	13.21	<0.001	Comp **1**: 66.96
			Comp **2**: 33.04
Variety	55.02	<0.001	Comp **1**: 59.63
			Comp **2**: 27.41
			Comp **3**: 12.96
(Drying) × (Variety)	29.39	<0.001	Comp **1**: 27.18
			Comp **2**–**6**: 72.82
Residuals	2.38	-	-

## Data Availability

Data is contained within the article and supplementary materials.

## Supplementary Materials

The following Supporting Information can be downloaded at: https://www.mdpi.com/article/10.3390/foods12051065/s1, Table S1: Volatile compounds identified in fresh and oven-dried (OD), sun-dried (SD), or sun-dried using a black plastic sheeting (SBPD) bulk (foratero (F) and CCN51 (C)) and fine-flavor (ETT103 (E) and LR14 (L)) cocoa, with the corresponding retention times (min), retention indexes (experimental and theoretical (NIST)), and ions of quantification.
