# Supplementary material for: Effect of Drying Technique on the Volatile Content of Ecuadorian Bulk and Fine-Flavor Cocoa
Source: Foods. 2023 Mar 2;12(5):1065. doi: 10.3390/foods12051065 (PMC10001102; doi:10.3390/foods12051065)
Supplement: Supplementary file 1 [file foods-12-01065-s001.zip › foods-2060119-supplementary.pdf]

Effect of drying technique on the volatile content of Ecuadorian bulk and fine-flavor cocoa

Cyntia Yadira Erazo Solorzano [1], Vincenzo Disca [2], JoséManuel Muñoz-Redondo [3], Diego Armando Tuárez García [1], Mónica Sánchez-Parra [3], Manuel Danilo Carrilo Zenteno [4], José Manuel Moreno-Rojas [3,5*] and Raquel Rodríguez-Solana [3,6*]

[1] Faculty of Industry and Production Sciences, State Technical University of Quevedo, Av. Walter Andrade, km 1.5 via Santo Domingo, C.P. 73, Quevedo 120301, Ecuador

[2] Dipartimento di Scienze del Farmaco, Università degli Studi del Piemonte Orientale "A. Avogadro", 28100 Novara, Italy

[3] Department of Agrifood Industry and Food Quality, Andalusian Institute of Agricultural and Fisheries Research and Training (IFAPA), Alameda del Obispo, Avda Menéndez Pidal s/n, 14004 Córdoba, Spain

[4] Instituto Nacional de Investigaciones Agropecuarias (INIAP), Estación Experimental Tropical Pichilingue (EETP), Departamento de Manejo de Suelos y Aguas (DMSA), km 5 vía Quevedo – El Empalme, Cantón Mocache, Quevedo 120313, Ecuador;

[5] Foods for Health Group, Instituto Maimónides de Investigación Biomédica de Córdoba (IMIBIC), 14004 Córdoba, Spain.

[6] MED–Mediterranean Institute for Agriculture, Environment and Development & CHANGE–Global Change and Sustainability Institute, Faculdade de Ciências e Tecnologia, Universidade do Algarve, Campus de Gambelas, 8005-139 Faro, Portugal

* Correspondence: josem.moreno.rojas@juntadeandalucia.es (J.M.M.-R.); raquel.rodriguez.solana@juntadeandalucia.es (R.R.-S.)

Supplementary information

Table S1. Volatile compounds identified in fresh, and oven-dried (OD), sun-dried (SD) or sun-dried using a black plastic sheeting (SBPD) bulk (foratero (F) and CCN51 (C)) and fine-flavor (ETT103 (E) and LR14 (L)) cocoa, with the corresponding retention times (min), retention indexes (experimental and theoretical (NIST)) and ions of quantification.

						**Fresh cocoa**				**OD**				**SD**				**SBPD**			
	**RT (min)**	**Compound**	**RI $_{experimental}$**	**RI $_{NIST}$**	**Ion$_Q$**	**F**	**C**	**E**	**L**	**F**	**C**	**E**	**L**	**F**	**C**	**E**	**L**	**F**	**C**	**E**	**L**
		**Aldehydes**																			
1	4.42	2-Methyl butanal	920	922	57	x	x	x	x	x	x	x	x	x	x	x	x	x	x	x	x
2	7.79	Hexanal	1087	1086	56	x	x	x	x												
3	13.99	(E)-2-Octenal	1443	1443	70	x		x	x												
4	14.38	3-(Methylthio)propanal	1471	1471	104	x	x	x												x	
5	14.54	Furfural	1483	1483	96							x	x								
6	15.36	Benzaldehyde	1545	1545	77	x	x	x	x	x	x	x	x	x	x	x	x	x	x	x	x
7	16.84	Benzacetaldehyde	1664	1663	91	x	x	x	x	x	x	x	x	x	x	x	x	x	x	x	x
8	20.07	α-Ethylidenbenzeneacetaldehyde	1955	1961	117	x	x	x	x	x	x	x	x	x	x	x	x	x	x	x	x
		**Alcohols**																			
9	7.12	2-Methylbut-3-en-2-ol	1056	1058	71	x	x	x	x												
10	8.2	2-Methyl-propanol	1106	1105	74											x					
11<br>12	8.72	3-Methyl-2-butanol +<br>2-pentanol	1131	1124<br>1130	73	x	x	x	x	x	x	x	x	x	x	x	x	x	x	x	x
13	10.35	3-Methyl-butanol	1213	1213	55	x	x	x	x	x	x	x	x	x	x	x	x	x	x	x	x
14	10.57	2-Hexanol	1226	1226	69	x	x	x	x												
15	10.63	2-Methyl-butanol	1229	1224	57		x			x	x	x	x	x	x	x	x	x	x	x	x
16	11.09	1-Pentanol	1255	1255	55	x	x	x	x												
17	12.24	2-Heptanol	1324	1324	55	x	x	x	x	x	x	x	x	x	x	x	x	x	x	x	x
18	12.6	4-Methyl-5-hexen-2-ol (probably)	1347	1344*/1348**	81	x	x	x	x	x	x	x	x	x	x	x	x	x	x	x	x
19	12.79	1-Hexanol	1360	1360	56	x	x	x		x		x				x					x
20	13.71	2-Octanol	1422	1422	55	x	x	x	x		x		x	x	x	x	x			x	x
21	15.07	2-Nonanol	1522	1521	69	x	x	x	x	x	x	x	x	x	x	x	x	x	x		x
22	15.38	2,3-Butanediol	1547	1544	57								x	x	x	x					
23	18.69	α-Phenylethanol	1826	1827	79	x	x	x	x	x	x	x	x	x	x	x	x	x	x	x	x
24	19.41	Benzyl alcohol	1893	1893	79	x	x	x	x	x	x	x	x	x	x	x	x	x	x	x	x
25	19.8	Phenylethyl alcohol<br>(2-Phenylethanol)	1930	1929	91	x	x	x	x	x	x	x	x	x	x	x	x	x	x	x	x
		**Acids**																			
26	14.24	Acetic acid	1461	1460	60					x	x	x	x	x	x	x	x	x	x	x	x
27	15.54	Propanoic acid	1556	1556	74					x	x	x	x	x	x	x	x	x	x	x	x
28	15.79	2-Methyl-propanoic acid	1578	1578	73					x	x	x	x	x	x	x	x	x	x	x	x
29	16.58	Butanoic_acid	1643	1642	60					x		x				x				x	x
30	17.23	2-/3-Methyl-butanoic acid	1685	1687/1685	74					x	x	x	x	x	x	x	x	x	x	x	x

	RT (min)	Compound	RI experimental	RI NIST	Ion$_Q$	Fresh cocoa				OD				SD				SBPD			
						F	C	E	L	F	C	E	L	F	C	E	L	F	C	E	L
		**Ketones**																			
31	5.62	2-Pentanone	986	985	86	x	x	x	x	x	x							x	x		
32<br>33	9.99	2-Heptanone +<br>5-methyl-2-hexanone	1188	1188<br>1188	58	x	x	x	x	x	x	x	x	x	x	x	x	x	x	x	x
34	11.7	2-Octanone	1290	1290	58	x	x	x	x	x	x	x	x	x	x	x	x	x		x	x
35	11.78	3-Hydroxy-2-butanone (acetoin)	1295	1295	88					x	x	x	x	x	x	x	x	x	x	x	x
36	12.97	2-Hydroxy-3-pentanone	1372	1370	57					x	x		x	x	x		x	x	x		x
37	13.23	2-Nonanone	1389	1389	58	x	x	x	x	x	x	x	x	x	x	x	x	x	x	x	x
38	16	3,6-Heptanedione (probably)	1595	1592***	57	x	x	x	x	x			x	x			x				x
39	16.96	Acetophenone	1674	1671	105	x	x	x	x	x	x	x	x	x	x	x	x	x	x	x	x
		**Esters**																			
40	4.14	Ethyl acetate	904	904	61						x	x	x	x	x	x	x	x	x	x	x
41	7.6	2-Pentyl acetate	1078	1074	87					x				x	x		x	x	x		
42	8.6	2-/3-Methylbutyl acetate	1125	1126	70					x	x	x	x	x	x	x	x	x	x	x	x
43	10.9	Ethyl hexanoate	1245	1245	88					x	x	x	x	x	x	x	x	x	x	x	x
44	11.3	1-Methylhexyl acetate<br>(2-heptanol acetate)	1273	1266	98					x	x	x	x	x	x	x	x	x	x	x	x
45	13.99	Ethyl octanoate	1443	1445	88					x	x	x	x	x	x	x	x	x	x	x	x
46	14.83	2,3-Butanedioldiacetate	1504	1501	87					x	x		x					x			
47	17.81	Benzyl acetate	1747	1747	108					x	x	x	x	x	x		x	x	x	x	x
48	18.43	Ethyl benzeneacetate	1802	1800	91					x	x	x	x	x	x	x	x	x	x	x	x
49	18.77	*β*-Phenylethyl acetate	1833	1833	104					x	x	x	x	x	x	x	x	x	x	x	x
50	18.78	Phenethyl pivalate	1833	1832	57						x	x		x	x	x			x		
51	19.03	Butyl benzoate	1857	1856	105	x	x	x	x	x	x	x	x	x	x	x	x	x	x	x	x
		**Terpenes**																			
52	9.32	*β*-Myrcene	1161	1161	93	x		x	x	x	x	x	x	x	x	x	x		x	x	x
53	10.16	D-Limonene	1202	1202	68	x							x	x		x	x				
54	10.78	Ocimene (isomers E and Z)	1236	1237	93	x		x	x		x	x	x			x	x			x	x
55	13.13	*γ*-Pyronene (1,3,5,5-tetramethyl-<br>1,3-cyclohexadiene)	1382	1406	121	x		x	x			x	x			x	x				x
56	14.09	Linalool oxide I	1450	1450	94	x	x	x	x	x	x	x	x	x	x	x	x		x	x	x
57	14.49	Linalool oxide II	1.478	1478	94	x	x	x	x	x	x	x	x	x	x	x	x	x	x	x	x
58	15.45	Linalool	1552	1552	71	x	x	x	x	x	x	x	x	x	x	x	x	x	x	x	x
		**Lactones**																			
59	16.49	Valerolactone	1635	1629	56						x	x	x			x				x	x
60	16.74	Butyrolactone	1656	1652	86					x	x	x	x	x	x	x	x	x	x	x	x
		**Miscelaneous**																			
61	13.59	trimethyl-Pyrazine	1414	1414	122					x	x	x	x	x			x	x			x
62	16.29	Isophorone	1618	1615	138					x		x									x
63	16.42	Benzonitrile	1629	1629	103					x		x				x				x	
64	19.28	*o*-Guaiacol	1881	1886	109					x								x			

Compounds tentative identified according to the linear retention index (RI) calculated in previous works: 4-methyl-5-hexen-2-ol, *Costa Castro Alves et al. [21] and **Li [22]; 3,6-Heptanedione, ***Raffo et al. [23]. RI: retention index; Ion Q: ion of quantification.
